# Influenza A virus hemagglutinin and neuraminidase act as novel motile machinery

**DOI:** 10.1038/srep45043

**Published:** 2017-03-27

**Authors:** Tatsuya Sakai, Shin I. Nishimura, Tadasuke Naito, Mineki Saito

**Affiliations:** 1 Department of Microbiology, Kawasaki Medical School, Kurashiki, Okayama 701-0192, Japan; 2 Research & Development, JamGuard, Minato, Tokyo 107-0052, Japan; 3 Research & Development, Sakura Academia Corp., Chiyoda, Tokyo 102-0074, Japan

## Abstract

Influenza A virus (IAV) membrane proteins hemagglutinin (HA) and neuraminidase (NA) are determinants of virus infectivity, transmissibility, pathogenicity, host specificity, and major antigenicity. HA binds to a virus receptor, a sialoglycoprotein or sialoglycolipid, on the host cell and mediates virus attachment to the cell surface. The hydrolytic enzyme NA cleaves sialic acid from viral receptors and accelerates the release of progeny virus from host cells. In this study, we identified a novel function of HA and NA as machinery for viral motility. HAs exchanged binding partner receptors iteratively, generating virus movement on a receptor-coated glass surface instead of a cell surface. The virus movement was also dependent on NA. Virus movement mediated by HA and NA resulted in a three to four-fold increase in virus internalisation by cultured cells. We concluded that cooperation of HA and NA moves IAV particles on a cell surface and enhances virus infection of host cells.

Influenza virus, Semliki Forest virus (SFV), and vesicular stomatitis virus (VSV) are enveloped viruses that are internalised into host cells by cellular endocytosis^1^. There are several pathways for virus entry such as clathrin-dependent endocytosis, caveolin-dependent endocytosis, and macropinocytosis^2^. An important aspect of virus infection is the manner through which viruses are targeted to endocytic machineries as entry pathways. There are two potential mechanisms for virus targeting. One is migration of viruses on cell surfaces to membrane domains with pre-existing endocytic machineries. The other is recruitment of endocytic machineries by viruses, such as clathrin cargo around the virus attachment site. Influenza virus uses the latter targeting mechanism. Influenza A virus (IAV) attaches to the surface membrane of a cultured cell, inducing assembly of clathrin cargo and endocytosis of the virus^3^,^4^. However, *in vivo*, influenza virus appears to require migration on the cell surface for targeting to the endocytic machinery. Human influenza virus infects epithelial cells in the respiratory tract, the surface of which is covered by abundant cilia. Because a cilium is thin with a diameter of ~0.25 μm and its inside is fully occupied by microtubule bundles^5^, there appears to be no space for endocytic invagination of the cell membrane. Endocytic machineries of a ciliated cell occupy the bases of cilia^6^. In addition, human influenza virus preferentially infects non-ciliated cells that are hidden by the cilia of neighbouring ciliated cells^7^. In contrast, avian influenza virus preferentially infects epithelial cells in the intestinal crypt at the bottom of the deep invagination of the intestine luminal surface^8^. An interesting aspect is the manner through which influenza viruses migrate to target cells or cellular domains. To address this issue, we investigated a novel motile mechanism of IAV driven by two viral membrane proteins, hemagglutinin (HA) and neuraminidase (NA).

Several mechanisms of virus movement have been reported previously. Simian Virus 40 (SV40) moves on an artificial membrane surface by lateral diffusion of its receptor in a lipid bilayer^9^. Murine leukaemia virus and VSV attach to cellular protrusions and rapidly move to the root of the protrusion by actin- and myosin-driving mechanisms, which is mediated by the viral receptor^10^. Both mechanisms of virus movement employ lateral movements of the viral receptor in the membrane lipid bilayer, which are essentially identical to the endocytosis of transferrin, low density lipoprotein (LDL), insulin, and epidermal growth factor (EGF) (Fig. 1a, left). Compared with these physiological ligands and other related viruses, binding of influenza virus HA to its receptor is remarkably weak. The dissociation coefficient (Kd) between HA and its receptor is 1.4–6.5 × 10^−3^ M^11^,^12^, which is 1 × 10^5^–10^11^-fold greater than the Kd of adhesive proteins on other viruses (e.g., the E2 protein of SFV and the G protein of VSV) or the Kd of several physiological ligands (Supplementary Table S1)^13^,^14^,^15^,^16^,^17^,^18^. Moreover, the HA-receptor binding half-life is 0.8–5.5 s^19^, whereas the half-life of influenza virus internalisation is 10–15 min^20^. Therefore, stable association of IAV with the cell surface requires multiple HA-receptor binding events. During these multiple binding events, individual HAs associate and dissociate with the same or neighbouring receptors. This receptor exchange of HA has been proposed to be “browsing” as a molecular mechanism of virus-induced haemagglutination^21^. In addition, a similar receptor exchange mechanism was recently observed in SV40^9^. On an artificial lipid bilayer containing receptor molecules, SV40 particles occasionally perform “tumbling” and “rocking” motions caused by the receptor exchange of SV40 viral protein 1. We hypothesised that the receptor exchange mechanism of HA directly acts as the driving mechanism to translocate virus particles across the cell surface (Fig. 1a, right). To test our hypothesis, we investigated fluorescently labelled IAV particles on glass surfaces with immobilised receptors, and observed migration of IAV across the surface by two distinct types of motions that were dependent on viral NA activity. Furthermore, blocking virus movement resulted in suppression of virus endocytosis by cultured cells. These data demonstrated a novel role of HA and NA in virus motility and entry.

## Results

### Direct observation of influenza virus movement

**Figure 1:**
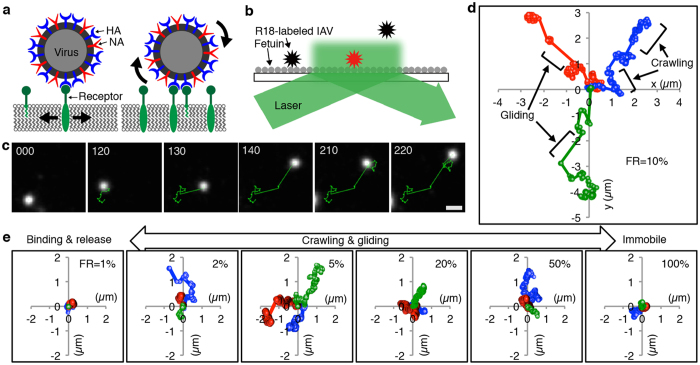
Influenza A virus movement. (**a**) Models for influenza virus movement via receptor lateral movement (left) or exchange of HA-receptor bindings (right). The virus was previously thought to move passively by lateral movement of the viral receptor, a sialoglycoprotein or sialoglycolipid (green), through the membrane lipid bilayer (left). This mechanism is identical to the endocytosis of transferrin, low density lipoproteins (LDL), insulin, and epidermal growth factor (EGF)^1^,^45^. However, compared with the bindings between these physiological ligands and their receptors, the binding between HA and its receptor is remarkably weak. We hypothesise that IAV movement occurs via HA-receptor exchanges where iterative association and dissociation between HAs and their receptors serve to translocate the virus particle across the surface (right). (**b)** Schematic of the setup to directly observe virus movements. To test our hypothesis, fetuin, functioning as a viral receptor, was fixed to the surface of glass coverslips. IAV (Aichi2) was labelled with R18, a lipophilic fluorescent probe. The use of TIRFM allowed observation of single viruses that were adjacent to the surface. (**c**) IAV movement on the fetuin-coated surface. The green line indicates the trajectory of the virus particle. The time (s) is indicated in the upper left of each panel. Scale bar = 1 μm. (**d**) Trajectories of three viruses. Virus positions per second are shown. The origin is defined as the location of the initial virus particle attachment. (**e**) Dependency of virus motility on receptor density. The trajectories of three viruses are shown in each panel. In (**d** and **e**), FR is the fetuin ratio which is the percentage of fetuin in the fetuin-albumin mixture used to coat the glass surface.

To investigate whether IAV movement occurs via the exchange of HA-receptor binding pairs, we coated the surfaces of glass coverslips with fetuin as a viral receptor and investigated the movement of the IAV strain Aichi/2/68(H3N2) (Aichi2) conjugated to a fluorescent probe, octadecyl rhodamine B (R18) on the fetuin-coated surfaces using total internal reflection fluorescence microscopy (TIRFM) combined with single particle tracking analyses (Fig. 1b). Using this method, we observed virus movements in two dimensions (Fig. 1c, Supplementary Movies S1 and S2). Generally, IAV movement can be described as gradual (crawling). However, occasional rapid (gliding) motion is observed (Fig. 1d). Virus gliding was also observed at a very low concentration of virus, indicating that gliding is different from one virus being released from the surface and then another virus immediately attaching to the surface. Virus movements were observed over a wide range of virus receptor densities (Fig. 1e). Glass surfaces were coated with a fetuin-albumin mixture in which the fetuin ratio (FR) varied from 0 to 100%. Although the actual fetuin density on the glass surface was unknown, the FR is the relative receptor density. The viruses clearly moved on surfaces coated with 2~50% fetuin. At low FRs (<2%), viruses did not move on the surface. Most virus particles immediately detached themselves from the surface without crawling or gliding. As the coating FR increased over 50%, the number of immobile viruses increased. Because virus motility was most active at a FR of around 10%, all subsequent virus movement experiments were performed on the glass surface coated with 10% fetuin.

### Quantitative analysis of virus movement

**Figure 2:**
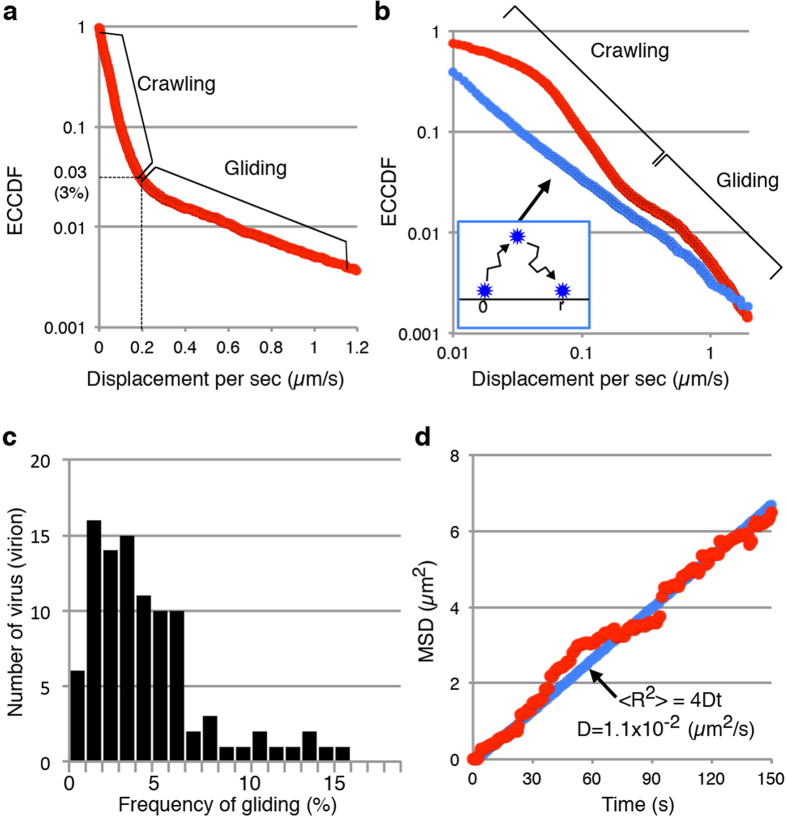
Quantitative analysis of IAV movements. (**a**–**d)** Movements of 100 Aichi2 virus particles were analysed. ECCDFs of virus displacement per second are presented on a semi-log plot (**a**) and a log-log plot (**b**). In both plots, ECCDFs are shown in red. A double exponential function in which the boundary is approximately 0.2 μm was observed in the semi-log plot of the ECCDF (**a**). ECCDF of simulated data (blue) assuming that the viruses float from the glass surface into water, move in a Brownian fashion, and return to the surface is shown for comparison with the ECCDF of actual virus movements (**b**). Individual virus motility was analysed (**c**). Gliding frequency was calculated by dividing the number of image frames recording gliding events by the number of total frames. The displacement of IAV particles was analysed, using a two-dimensional random diffusion (random walk) model (**d**). The mean square displacement* (MSD) of viruses (red) was fitted by the equation for two-dimensional random diffusion (blue): 〈R^2^〉 = 4Dt, where 〈R^2^〉 is the MSD of viruses and D is the diffusion coefficient. This fitting resulted in D = 1.1 × 10^−2^ μm^2^/s. *Note: the displacement (R) of the MSD in (**c**) is taken from the position where the virus particles initially attach, which is not the same as the displacement per second shown in (**a** and **b**).

Quantitative analyses of 100 virus particles were undertaken to adequately characterise the crawling and gliding motions of Aichi2 virus (Fig. 2a–d). Using the empirical complementary cumulative distribution function (ECCDF), frequencies of crawling and gliding were analysed (Fig. 2a and b). The ECCDF is defined as


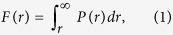


where *F(r)* is the ECCDF of *r, r* is the displacement of virus particles per second, and *P(r)* is the frequency function of *r*. As the displacement elongated, its appearance frequency became lower. Therefore, virus motions with long displacements were difficult to analyse by the normal frequency function, *P(r)* (Supplementary Fig. S1). ECCDF is useful to estimate whether noisy tails of frequency functions obey power or exponential laws, because ECCDF is the integral of the frequency function. The ECCDF curve consisted of two different phases and the boundary displacement between the two phases was 0.2 μm per second (Fig. 2a). Here, we defined motion shorter than 0.2 μm per second as crawling and motion longer than 0.2 μm per second as gliding. Because ECCDF analysis could not distinguish crawling and stationary phases, the lower limit of crawling motion was unknown. Moreover, the exact speed of gliding remained unknown. Even higher speed image recording every 0.1 second could not trace viruses while gliding, indicating that gliding events were completed within 0.1 second. However, ECCDF analysis showed that the gliding frequency was 3%, and the remaining 97% included crawling and stationary phases (Fig. 2a). The frequencies observed for crawling and gliding suggest that both motions are different from the process in which a virus floats away from a fetuin-coated glass surface, moves three-dimensionally in a Brownian fashion in the surrounding water, and then returns to the glass surface. Figure 2b shows a simulated plot (blue), which is a power function unlike the experimental data (red), suggesting that IAV particles do not float in water when they are crawling or gliding.

To test for heterogeneity of virus motility, we examined the gliding frequency of individual viruses (Fig. 2c). All viruses showed similar results and no virus had considerably high or completely abolished gliding activity.

The mean square displacement (MSD) of IAV particles indicated that the viruses moved two-dimensionally in a random fashion (random walk) (Fig. 2d) that is different from the three-dimensional Brownian movement. The diffusion coefficient for the random walk of the viruses investigated in the current study was 1.1 × 10^−2^ μm^2^/s, which is similar to the previously reported values for the lateral diffusion of transferrin, LDL, insulin, and EGF receptors (Fig. 2d and Supplementary Table S2)^22^,^23^,^24^. Our results indicate that IAV crawling and gliding are able to deliver virus particles to endocytosis regions without the lateral movement of receptors in the membrane lipid bilayer.

### Role of NA in virus movement

**Figure 3:**
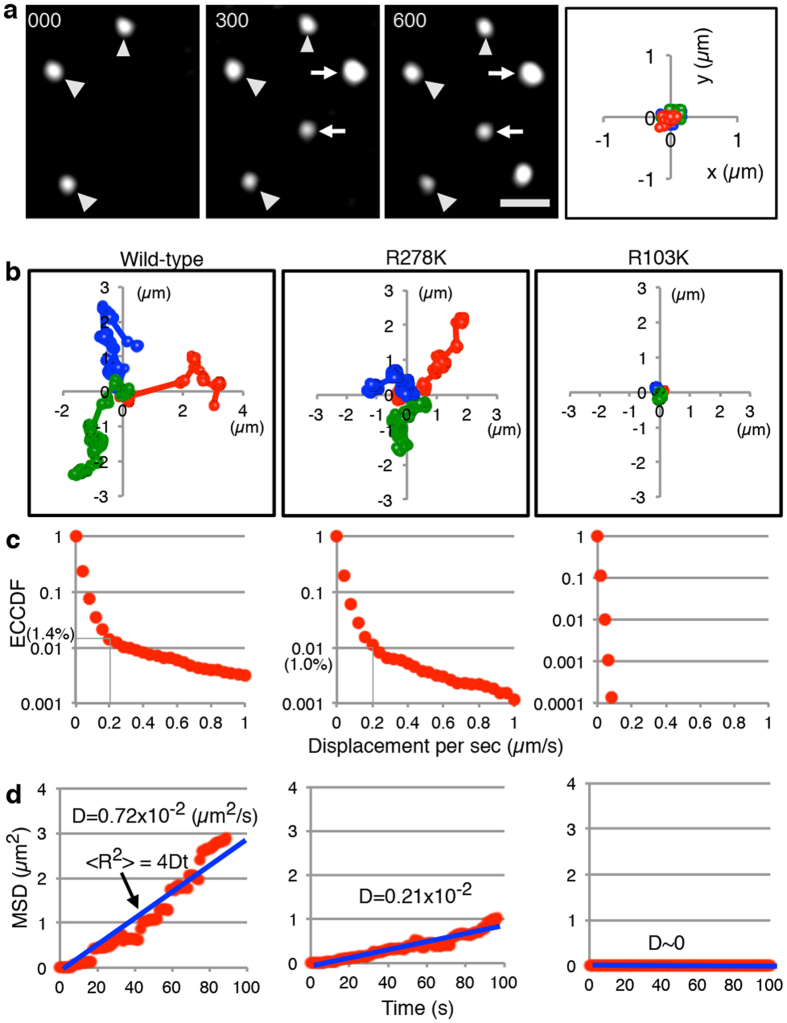
NA contribution to virus motility. (**a**) Aichi2 virus motility in the presence of zanamivir. Arrowheads indicate virus particles that did not move for more than 10 min, while arrows indicate virus particles that did not move for more than 5 min. The time (s) is indicated in the upper left of each panel. Scale bar = 1 μm. The trajectories of three viruses over a 10-min period are shown in the right panel. (**b–d**) Wild-type and mutant PR8 virus motility. (**b**) Trajectories of three virus particles per virus type. (**c**) Semi-log plots of ECCDFs. A double exponential function in which the boundary was approximately 0.2 μm was observed for the wild-type and NA R278K viruses. (**d**) MSDs of each virus were fitted by the equation of two dimensional random diffusion (blue line), 〈R^2^〉 = 4Dt, and the diffusion coefficients were calculated. For (**c** and **d)** 50 particles per virus type were analysed.

Generally, influenza virus NA is thought to mediate progeny virus budding from host cells^25^. Nevertheless, NA inhibitors reportedly suppress virus infection, indicating a major role of NA in infection^26^,^27^,^28^. However, the specifics of this functional role remained unclear. We hypothesised that NA contributes to the movement of virus particles toward endocytosis regions. To test this hypothesis, we examined the effect of a NA inhibitor, zanamivir, on Aichi2 virus crawling and gliding motions on fetuin-coated surfaces. This NA inhibitor completely blocked virus crawling and gliding, resulting in the immobilisation of virus particles on the glass surface (Fig. 3a, and Supplementary Movie S3). This finding indicates that NA contributes to the initiation of crawling and gliding motions.

To elucidate the role of NA in the observed crawling and gliding motions in more detail, we examined this process with recombinant viruses harbouring NA mutations that were generated by reverse genetics. Previously, amino acid substitution of arginine residues to lysine residues at the NA catalytic site has been reported to decrease NA enzymatic activity^29^. We introduced mutations into the catalytic site of NA in the A/Puerto Rico/8/34(H1N1) (PR8) virus and created two NA mutant (R103K and R278K) viruses. The wild-type and NA R278K viruses were able to move by crawling and gliding on the glass surfaces coated with immobilised fetuin, and their movements were similar to the movement exhibited by the Aichi2 virus (Fig. 3b, Supplementary Movies S4 and S5). In contrast, the NA R103K virus did not exhibit any movement (Fig. 3b and Supplementary Movie S6). Results from quantitative analysis of virus movement showed that the motile activity of NA R278K virus was lower than that of the wild-type virus (Fig. 3c and d). Specifically, the gliding frequency of the NA R278K virus (1.0%) was lower than that of the wild-type virus (1.4%) (Fig. 3c). Furthermore, the tail of the ECCDF curve for the NA R278K virus decreased more rapidly than that of the wild-type virus, indicating that its gliding length was shorter than that of the wild-type virus (Fig. 3c). Consequently, the diffusion coefficient of the NA R278K virus (0.21 × 10^−2^ μm^2^/s) was 3.4 times smaller than that of the wild-type virus (0.72 × 10^−2^ μm^2^/s) (Fig. 3d). The NA enzymatic activity of total viral proteins (i.e., for a virus particle) of the NA R278K virus was 34.5% relative to that of the wild-type virus (Table 1). The lower NA activity resulted in a decrease in the diffusion coefficient of virus movement (i.e., limited motility) from the diminished virus gliding frequency and gliding length, indicating that NA contributes to both initiation and elongation of the gliding movement. Whereas the NA enzymatic activity of total viral proteins of the NA R103K virus was similar to that of the NA R278K virus, the NA R103K virus lacked motility and was markedly different from the NA R278K virus (Table 1, Fig. 3b, and Supplementary Movies S5 and S6). However, the expression level of the NA R103K molecule was 40.6% relative to that of the wild-type NA, whereas that of the NA R278K molecule was 83.2% (Supplementary Fig. S2 and Table 1). Importantly, these data suggest that initiation of virus crawling and gliding appears to require both adequate NA activity and a certain density of NA molecules on the virus surface.

### Virus movement on cell surfaces

**Figure 4:**
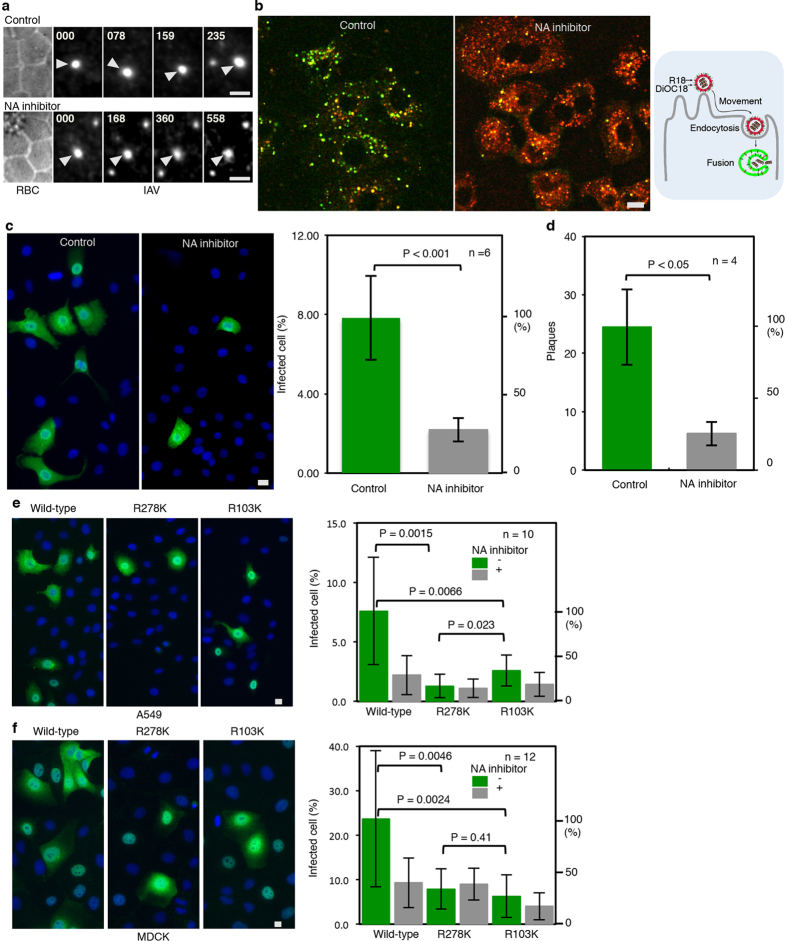
IAV movement on cell surfaces and infection inside of cells. (**a)** IAV movement on erythrocytes in the absence or presence of the NA inhibitor zanamivir. The left panels (RBC) show bright field images of erythrocytes fixed to a glass coverslip. The remaining panels (IAV) are florescence images of viruses on erythrocytes. Arrows indicate identical virus particles. Time (s) is indicated in the upper left of each panel. Scale bar = 5 μm. (**b)** Virus-endosome fusion assay. A549 cells were inoculated with R18- and DiOC18-labelled Aichi2 viruses in the presence (NA inhibitor) or absence (control) of zanamivir. A change from red to green fluorescence indicates virus-endosome fusion. A schematic of virus-endosome fusion detection is illustrated in the right panel. Scale bar = 10 μm. (**c)** Indirect immunofluorescence (IIF) assays of IAV protein synthesis. After inoculation with Aichi2 viruses in the presence (NA inhibitor) or absence (control) of zanamivir, the cells were washed and incubated in zanamivir-free medium for 6 h. Cells producing viral proteins and cell nuclei fluoresced green and blue, respectively. Scale bar = 10 μm. Percentages of cells producing viral proteins were calculated and are summarised in the right histogram. The means ± standard deviations (SD) of six paired experiments are shown. Unpaired *t*-tests were conducted to determine the *p*-values. *p* < 0.001 was considered as statistically significant. (**d)** Plaque forming assay. Following infection with Aichi2 viruses, the cells were washed and overlaid with zanamivir-free agarose and incubated for 24 h, after which the plaques were counted. The means ± SD of four paired experiments are shown. Unpaired *t*-tests were conducted to determine the *p*-values. *p* < 0.05 was considered as statistically significant. (**e**,**f**) IIF assays of wild-type and NA mutant PR8 virus infection of A549 and MDCK cells. A549 or MDCK cells (1 × 10^5^ cells) were infected with purified wild-type or mutant viruses (corresponding to 5 ng viral protein) in the absence or presence of zanamivir. Infection, incubation, staining, and analysis were performed as described in **c**. Images show cells in the absence of zanamivir. Scale bar = 10 μm.

We next used TIRFM to examine Aichi2 virus movement on human erythrocytes. We found that viruses moved around the cell surface (Fig. 4a and Supplementary Movie S7). The NA inhibitor zanamivir adversely affected virus movement on the cell surface, resulting in movements similar to those of zanamivir-treated viruses on fetuin-coated surfaces (Fig. 4a and Supplementary Movie S8). This result indicates that IAV particle movement on erythrocytes is driven by the HA-receptor exchange mechanism that is accelerated by NA. In the presence of an NA inhibitor, a previous report showed that IAV particles embed in the erythrocyte membrane^28^. Presumably, abolished NA activity induces an increase in HA-receptor binding cross-bridges around the virus particle, causing the virus to eventual embed in the membrane. More importantly, NA activity that restricts the number of HA-receptor cross-bridges might contribute to virus movement on an actual cell surface rather than virus movement on a receptor-coated glass surface.

### Biological significance of virus movement

NA inhibitors such as zanamivir and oseltamivir suppress virus infection^26^,^27^,^28^. Our observation of NA-dependent IAV movement on cells implies that virus movement contributes to endocytosis of IAVs. To determine whether virus movement via HA-receptor exchange contributes to their delivery to endocytosis regions, we examined virus fusion inside of cells and the effects of zanamivir on such fusion. Following endocytosis, viruses fuse with the endosomal membrane^1^,^30^. To detect virus-endosome fusion, we used an imaging technique in which virus-endosome fusion induces fluorescence colour changes from red to green in viruses labelled with two different fluorescent probes, R18 and 3,3′-dioctadecyloxacarbocyanine (DiOC18) (Fig. 4b)^31^. We also applied this technique to evaluate the effects of zanamivir on virus-endosome fusion in cultured A549 cells. In the presence of zanamivir, cells were inoculated with R18- and DiOC18-labelled Aichi2 viruses for 30 min at 37 °C. Following infection, virus particles predominantly fluoresced red (Fig. 4b), whereas in the absence of the inhibitor, virus particles fluoresced green. These observations indicate that the NA inhibitor suppresses virus-endosome fusion. Because zanamivir blocked virus movements by HA-receptor exchange (Fig. 3a), this finding suggests that virus movement may accelerate virus internalisation.

To evaluate the inhibitory effect of zanamivir on virus internalisation, we infected A549 cells with Aichi2 virus and examined viral protein synthesis and plaque formation. The NA inhibitor decreased the percentage of cells producing viral proteins and virus plaque formation to approximately 25% compared with untreated controls (Fig. 4c,d). We also obtained consistent results from a similar analysis of the virus infectivity of a PR8 NA mutant, the NA R103K mutant virus, which completely lacked motility (Fig. 3b). The infectivity of this virus in A549 cells and MDCK cells was 34% and 27%, respectively, of the wild-type virus infectivity (Fig. 4e,f). These results indicate that virus movement by the HA-receptor exchange mechanism may accelerate virus internalisation by three to four fold.

Analysis of NA mutant virus infectivity indicated that limited motility was ineffective for virus infection. The NA R278K mutant virus had suppressed motility (Fig. 3b). Infectivity of this mutant virus in A549 and MDCK cells was 17% and 33%, respectively, of the wild-type virus infectivity (Fig. 4e,f). The infectivity of the NA R278K mutant virus was slightly lower than that of the wild-type virus in the presence of the NA inhibitor in both cell types (Fig. 4e,f). This result indicated that the suppressed NA activity of the mutant virus did not contribute to virus infection. Furthermore, infectivity of this mutant virus in the presence of NA inhibitor was not different from the infectivity in the absence of the inhibitor, confirming that NA activity of this mutant virus was ineffective for virus infection. These results suggest that all types of virus movements are not necessarily effective for virus infection and there is an optimal movement pattern for virus infection.

## Discussion

**Figure 5:**
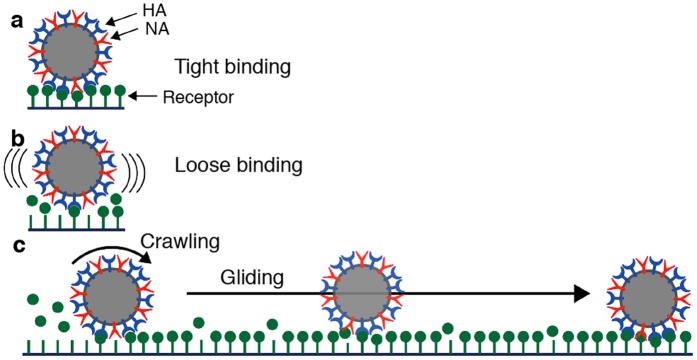
Models for crawling and gliding movements of IAV particles on fetuin-coated surfaces. (**a**) Immediately after a virus attaches to the fetuin-coated surface, multiple HA-receptor interactions tightly bind the virus to the surface. (**b**) Viral NA degrades receptors near the virus attachment site. The HA-receptor pairs thus decrease in number, and the virus becomes loosely attached to the cell surface. (**c)** Using iterative association and dissociation of HA-receptor interactions as a driving mechanism, the loosely attached virus performs crawling and gliding movements until reaching a site where multiple HA-receptor pairs can form and, again, tightly bind the virus to the surface.

A significant conclusion of this study is that IAV can move using the HA-receptor exchange mechanism (Fig. 1). The virus movement included crawling and gliding motions (Figs 1 and 2). Virus NA contributed to initiation of the crawling and gliding as well as to elongation of the gliding (Fig. 3). Based on these results, we devised a model (Fig. 5) describing the crawling and gliding mechanism of IAV on the fetuin-coated surface. In the first step (Fig. 5a), immediately following virus attachment to the fetuin-coated surface, multiple receptor molecules tightly bind virus particles to the surface via HA, forming HA-receptor pairs. When there are enough NA molecules near the virus attachment site, bound HA-receptor pairs decrease in number and the virus becomes loosely attached to the cell surface (Fig. 5b). Loosely attached virus particles on the fetuin-coated surface are hit by water molecules and forced three-dimensionally from all directions. The interaction of HA and receptors keeps the virus particle on the cell surface, and their iterative association and dissociation translocate the virus on the surface two-dimensionally. In this situation, the impact of water molecules and receptor exchange of HA act as a driving force and direction control machinery, respectively, for virus movement. The loosely attached virus continues to move until it reaches a site where enough HA-receptor pairs can form, again tightly binding the virus to the surface (Fig. 5c). Because there are two types of virus motion, crawling and gliding, there are at least two loosely bound states with different affinities of the virus particle for receptors. When the loosely attached virus with a relatively higher affinity is captured by receptors that are close (<0.2 μm), it performs a crawling motion. When the loosely attached virus with a relatively lower affinity is captured by receptors that are further apart (≥0.2 μm), it performs a gliding motion. While the virus particle is gliding, NA degrades the receptors, limiting the number of HA-receptor interactions, prolonging the loose binding state with the lower affinity, thereby increasing the gliding length.

Results of infection experiments using Aichi2 virus in the presence of the NA inhibitor or the PR8 mutant virus lacking NA activity (NA R103K) indicated that virus movement by this HA-receptor exchange mechanism accelerated virus entry into cultured cells by three to four fold (Fig. 4b–f). This effect probably underestimates the contribution of virus movement to viral infections *in vivo*. Reportedly, NA inhibitors decrease the levels of infection in primary cultured respiratory epithelial cells by 20–500 fold^26^. Although the effect of the NA inhibitor on cultured epithelia may be caused by various factors such as the mucus layer containing sialoglycoproteins, virus movement via HA-receptor exchange might be an important factor. In cultured epithelia, influenza viruses preferentially infect non-ciliated cells^7^. Because the non-ciliated cells are surrounded by ciliated cells and the surface of non-ciliated cells is covered with cilia, intercellular migration from ciliated to non-ciliated cells is necessary for virus infection. Whereas lateral movement of virus receptors along the cell membrane cannot enable intercellular virus migration, virus movement via HA-receptor exchange could explain how intercellular migration occurs in cultured epithelia. Virus movement mediated by HA-receptor exchange likely plays a crucial role in the infection of epithelial cells in the respiratory tract.

In addition to intercellular migration, virus movement via HA-receptor binding exchange confers another potential advantage for virus infection compared with lateral movement of a receptor along the cell membrane. When using HA-receptor binding exchange, a virus would be able to move even if bound to immobile receptors. Attachment to immobile receptors would occur when membrane proteins and lipids are restricted from free lateral diffusion in lipid bilayers by membrane skeletons, membrane rafts, and cytoskeletons^22^,^32^,^33^. IAV receptors are sialoglycoproteins and sialoglycolipids. Therefore, unlike VSV and SFV, IAV cannot selectively attach to receptor molecules mediating endocytosis (Supplementary Table S1). To escape from immobile receptors, IAV might need to move via the HA-receptor exchange mechanism. In addition to influenza viruses, several paramyxoviruses and coronaviruses, such as parainfluenza, Sendai viruses, and bovine coronavirus, employ sialoglycoproteins and sialoglycolipids as viral receptors^34^,^35^. Therefore, these viruses cannot selectively attach to receptor molecules that undergo endocytosis. To escape from immobile receptors, these viruses might use the receptor exchange mechanism.

We further hypothesised that altering the ratio of crawling and gliding motions might influence IAV host specificity. IAV host specificity is dependent on virus receptor types. Human influenza HAs attach to oligosaccharides with terminal sialic acids (SA) linked to neighbouring galactoses (Gal) via α2–6 linkages (SAα2–6 Gal), whereas avian virus HAs attach to oligosaccharides via SAα2–3 Gal linkages^36^. However, human influenza HAs attach to SAα2–3 Gal with substantial affinity, and the dissociation coefficients of HA from SAα2–6 Gal and SAα2–3 Gal are 2.1 mM and 3.2 mM, respectively^11^. In addition to the receptor type, the functional balance between HA affinity and NA catalytic activity is important for virus infectivity of specific host cells^37^,^38^,^39^. The IAV movement pattern depended on receptor density and NA activity (Figs 1d,e and 3). Virus crawling and gliding presumably depend on the balance between HA affinity and NA activity. IAV might change virus movement patterns by modifying HA and NA to adapt to host cell surface properties, such as shape, viral receptor density, or composition, thus resulting in maximal infectivity. In the future, further characterisation of human influenza virus movement patterns could help to identify virus strains that are a high risk to public health.

Our study demonstrated that IAV movement across the host cell surface was coordinated by its HA and NA proteins. This novel motility mechanism enables efficient access to target cells or cellular surface domains with high endocytosis activity. Until now, it was generally accepted that no viruses, including influenza, possess motile machinery. Our findings confirmed the presence of motile machinery employing HA and NA molecules in IAV. This virus motility mechanism is different from those known to exist in bacterial and eukaryotic cells. Further studies on this virus motility mechanism, particularly quantitative analysis of HA affinity and NA activity, might lead to a new paradigm for molecular mechanisms and energy transfer mechanics. Furthermore, systematic analysis and categorisation of the movement patterns of various viral strains could provide valuable information to understand the infectivity and host specificity of influenza viruses.

## Methods

### Viruses, cells and regents

The influenza Aichi2 virus was prepared from allantoic fluids of infected eggs at 2 days post-infection (dpi). The influenza PR8 wild-type and mutant viruses were generated by reverse genetics as described previously^40^. Additional information about plasmid construction and reverse genetics is provided in the Supplementary Methods. These viruses were similarly inoculated into eggs, except the inoculum of the NA R103K virus contained 1.5 mU *Clostridium perfringens* NA (Sigma-Aldrich, St. Louis, MO, USA). These viruses were isolated from allantoic fluids at 3 dpi. All viruses were purified by differential centrifugation and sucrose density gradient centrifugation.

A549, MDCK, and 293T cells were subcultured using Dulbecco’s modified Eagle’s medium (DMEM, Nissui, Tokyo, Japan) containing 10% fetal calf serum. Zanamivir was provided by GlaxoSmithKline Research and Development Ltd. (Stevenage, UK).

### Imaging virus movement

IAVs were labelled with R18 (Molecular Probes, Eugene, USA) as follows. Purified virus (100 μg viral proteins) was suspended in 1 ml phosphate-buffered saline (PBS; Nissui, Tokyo, Japan), and R18 was dissolved in ethanol to a final concentration of 100 μM. The R18 solution (10 μl) was added to the virus suspension and mixed vigorously. The reaction mixture was then gently mixed for 1 h at room temperature and subsequently passed through a filter (0.22-μm pore size).

For imaging analyses of virus movements, the glass bottoms of culture dishes (Iwaki, Tokyo, Japan) were coated with bovine fetuin (0.1 mg/ml, Sigma-Aldrich) and bovine serum albumin (0.9 mg/ml, Sigma-Aldrich) dissolved in PBS. In experiments to determine the dependency of virus motility on receptor density, the glass bottoms of culture dishes were coated with fetuin (0–1.0 mg/ml) and albumin (0–1.0 mg/ml) dissolved in PBS. Total protein concentrations of fetuin and albumin were adjusted to 1 mg/ml. The coating FR was defined as


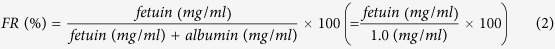


The glass surface was incubated for 1 h at room temperature and then washed once with PBS and twice with distilled water to remove any unbound fetuin. Culture dishes were dried at room temperature for 30 min. This cycle of coating and drying was repeated three times. To examine virus behaviour, fluorescently labelled viruses diluted approximately 100–1,000 times by PBS were added to the fetuin-coated glass surfaces and observed by TIRFM (Olympus, Tokyo, Japan) using a 150× (numerical aperture = 1.45) objective lens. Usually, fluorescence images were acquired every second for a total duration of 15 min. For high speed imaging, fluorescence images were acquired every 0.1 second for a total duration of 1 min.

To explore virus movement on a cell surface, a monolayer of erythrocytes was prepared on the bottom surface of glass dishes. Human erythrocytes were washed three times with PBS and resuspended in PBS to a final concentration of 1% (v/v). The erythrocyte suspension was allowed to attach to poly-L-lysine-coated glass at 4 °C for 15 min. The glass was then washed with PBS three times to remove unbound erythrocytes. The movement of fluorescently labelled viruses on erythrocyte surfaces was observed by TIRFM as described above.

### Analysis of virus movement

Images were analysed with Particle Tracker of ImageJ software (http://rsb.info.nih.gov/ij/)^41^. The positions (x and y coordinates) of virus particles were determined as the centres of fluorescence brightness. To test the stability and accuracy of our apparatus, time-lapse images of fluorescent latex spheres (100 nm, Molecular Probes) fixed on a cover slip were acquired. The displacement of fixed spheres was observed to vary by less than 20 nm for 1 s (corresponding to one image frame), which is comparable with published values from previous studies^42^,^43^.

We analysed the displacements per second of IAV particles using the ECCDF that is defined as Eq. (1). ECCDF is useful when estimating whether noisy tails of frequency functions obey power or exponential laws because ECCDF is the integral of the frequency function.

The displacement of IAV particles was analysed using a two-dimensional random diffusion (random walk) model. The MSD of viruses was fitted to the equation of two-dimensional random diffusion:


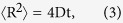


where R is the displacement of virus, which is obtained from the position where the virus particles initially attaches, 〈R^2^〉 is the MSD of viruses, D is the diffusion coefficient, and t is time.

### NA activity and expression assays

The NA activity of each purified virus was measured by a fluorometric assay using the fluorogenic substrate 2′-(4-methylunberiferyl)-α-D-*N*-acethylneuraminic acid (MUNANA; Sigma-Aldrich)^29^,^44^. The NA assay was performed in 33 mM morpholineethanesulfonic acid (pH 7.4) and 4 mM CaCl_2_ with MUNANA at a final concentration of 500 μM. The reaction mixtures (20 μl) were incubated at 37 °C for 30 min and stopped by the addition of 130 μl stop solution (14 mM NaCl and 0.1 M glycine in 25% ethanol; pH 10.7). Fluorescence of the released 4-methylunberiferon was measured with a Varioskan Flash fluorescence microplate reader (Thermo Fisher Scientific) using excitation and emission wavelengths of 355 and 460 nm, respectively.

NA expression levels were examined by western blot analysis. Viral proteins were separated by sodium dodecyl sulfate gel electrophoresis (SDS-PAGE) in a 5–20% gradient gel and then transferred to a nitrocellulose membrane. The membrane was stained with anti-NA mouse immunoglobulin G (IgG) and anti-M1 rabbit IgG (GeneTex, San Antonio, TX), followed by staining with fluorescently labelled secondary antibodies, IRDye680CW goat anti-mouse IgG and IRDye800CW goat anti-rabbit IgG (LI-COR, Lincoln, NE). Fluorescent intensities of NA and M1 bands were measured with an infrared imaging system Odesay CLx (LI-COR). Relative NA expression levels were evaluated using the fluorescence intensities of NA bands standardised to the intensities of M1 bands.

### Imaging virus fusion

Aichi2 virus was labelled with DiOC18 (Molecular Probes) and R18 using the same protocol described above for R18 labelling except that a probe mixture (6 μl) containing both DiOC18 (33 μM) and R18 (67 μM) was used instead of R18 alone. A549 cells cultured in glass dishes were incubated with fluorescently labelled viruses in the absence or presence of zanamivir (5 μM) for 30 min at 37 °C and then washed with PBS. Imaging was conducted using the dual wavelength imaging technique^31^. Briefly, the dish was placed on the stage of a confocal microscope (TCS-SP2 MP, Leica, Wetzlar, Germany) equipped with a 100× (NA = 1.4) objective lens. Cells were scanned using an argon-krypton laser (488 nm), and two ranges of emitted light (510–525 nm, green fluorescence; 575–640 nm, red fluorescence) were simultaneously detected before the acquired images were merged.

### Indirect immunofluorescence assays

To examine Aichi2 virus infectivity, A549 cells were cultured overnight on a 24-well pate (1 × 10^5^ cells/well) and infected with Aichi2 viruses in the presence or absence of zanamivir (5 μM) for 30 min at 37 °C. The cells were washed five times with DMEM to remove residual zanamivir and then incubated in serum-free medium (VP-SFM, Invitrogen, Carlsbad, CA, USA) for 6 h at 37 °C for virus production. The cells were then fixed with 1% paraformaldehyde in PBS, and viral antigens were detected by indirect immunostaining using a rabbit anti-human influenza A virus antibody (Takara; Kusatsu, Japan) and Alexa Fluor 488-labelled goat anti-rabbit IgG (Abcam; Cambridge, UK). After staining with the secondary antibody, cell nuclei were counterstained with 4′,6-diamidine-2′-phenylindole dihydrochloride. Using an image screening system (ImageXpress Micro, Molecular Devices; Tokyo, Japan), more than 1 × 10^4^ cells were examined in each experiment, and cells producing viral proteins were counted.

To examine PR8 wild-type and mutant virus infectivity, A549 and MDCK cells were cultured overnight on a 24-well pate (1 × 10^5^ cells/well) and then infected with purified wild-type or mutant viruses (corresponding to 5 ng viral proteins) in the presence or absence of zanamivir (5 μM) for 30 min at 37 °C. The cells were washed five times with DMEM and then incubated in VP-SFM for 6 h at 37 °C for virus production. After the incubation, staining and counting of cells were performed by the procedure described above.

### Plaque assays

Confluent A549 monolayers in six-well plates were infected with Aichi2 viruses in the presence or absence of zanamivir (5 μM) for 30 min at 37 °C. The cells were washed five times with DMEM to remove residual zanamivir and overlaid with VP-SFM containing 0.55% (w/v) agarose and 1 μg/ml acetylated trypsin. Plaques were counted at 1 dpi.

## Additional Information

**How to cite this article:** Sakai, T. *et al*. Influenza A virus hemagglutinin and neuraminidase act as novel motile machinery. *Sci. Rep.* **7**, 45043; doi: 10.1038/srep45043 (2017).

**Publisher's note:** Springer Nature remains neutral with regard to jurisdictional claims in published maps and institutional affiliations.

## Supplementary Material

Supplementary Movie S1

Supplementary Movie S2

Supplementary Movie S3

Supplementary Movie S4

Supplementary Movie S5

Supplementary Movie S6

Supplementary Movie S7

Supplementary Movie S9

Supplementary Information

## Figures and Tables

**Table 1:** NA activity and expression of wild-type and NA mutant viruses.

Influenza virus		NA activity (mean ± SD)^a^ nmol·min^−1^·μg^−1^ (%)	NA expression (mean ± SD)^b^ %	NA activity^c^ %
A/PR/8/1934(H1N1)	Wild-type	1.48 ± 0.02 (100 ± 1.3)	100	100
	NA R278K	0.52 ± 0.01 (35.4 ± 0.5)	83.2 ± 14.1	42.7
	NA R103K	0.46 ± 0.01 (31.5 ± 0.9)	40.6 ± 9.6	72.4
A/Aichi/2/1968(H3N2)		6.97 ± 0.24 (478.5 ± 16.2)	ND	ND

^a^NA activity of total viral proteins. Data are expressed as the mean ± SD (n = 4). ^b^NA expression levels standardised to M1. Data are expressed as the mean ± SD (n = 3). ^c^NA activity of NA molecules calculated by dividing NA activity of total viral proteins by the NA expression level. ND indicates not determined.
