# Supplementary material for: Influenza A virus hemagglutinin and neuraminidase act as novel motile machinery
Source: Sci Rep. 2017 Mar 27;7:45043. doi: 10.1038/srep45043 (PMC5366856; doi:10.1038/srep45043)
Supplement: Supplementary Information [file srep45043-s9.pdf]

Supplementary Information for:

Influenza A virus hemagglutinin and neuraminidase act as novel motile machinery

Tatsuya Sakai, Shin I. Nishimura, Tadasuke Naito, and Mineki Saito

Supplementary Methods

Supplementary Figures S1 and S2

Supplementary Tables S1 and S2

Supplementary Movies S1–S8

Supplementary Methods

Plasmid construction and reverse genetics. Plasmids for PR8 mutant viruses were provided by Yoshihiro Kawaoka (University of Tokyo). Mutations in the NA catalytic site (R103K or R278K) were introduced into the plasmid containing the wild-type NA coding sequence (pPol1-NAwt) by site-directed mutagenesis. To construct the plasmid containing the NA R103K mutant-coding sequence (pPol1-NA-R103K), two DNA fragments corresponding to the NA-coding sequence were amplified by PCR using 5′-CGTATTGGTCTCAGGGAGCRAAAGCAGG-3′ (Bsa-Uni-F) and 5′-CGTTTTTGTCATAAAAGAGCCCTTTATT-3′ or 5′-CGATATGGTCTCGTATTAGTAGAAACAAGG-3′ (Bsa-Uni-R) and 5′-AATAAAGGGCTCTTTTATGACAAAAACG-3′ primers and pPol1-NAwt as the PCR template. To construct pPol1-NA-R278K, two DNA fragments corresponding to the NA-coding sequence were amplified by PCR using Bsa-Uni-F and 5′-ATGTGTGTGTGCAAAGACAATTGGCAT-3′ or Bsa-Uni-R and 5′-ATGCCAATTGTCTTTGCACACACACAT-3′ primers. Full length NA (R103K) and NA (R278K) genes were amplified by PCR using Bsa-Uni-F and Bsa-Uni-R

primers. PCR products were digested with *Bsa*I and cloned into a *Bsm*BI-digested pPol1 plasmid. The appropriate plasmids were transfected into 293T cells, and recombinant viruses were recovered as described previously[40]. Transfected 293T cells were incubated at 37 °C in OPTI-MEM (Thermo Fisher Scientific, Yokohama, Japan), and then the supernatant was harvested at 48 h post-transfection. The NA R103K virus was generated in the presence of 200 µl/ml of *C. perfringens* NA.

Supplementary Figures

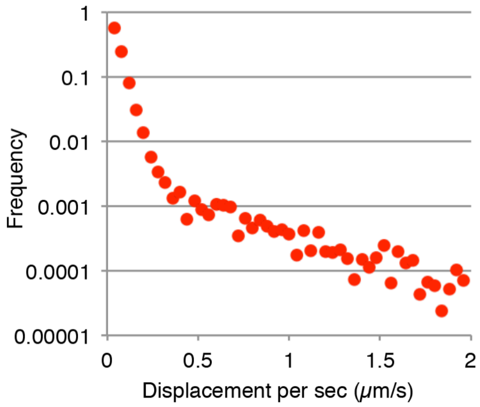

Supplementary Fig. S1. Frequency distribution of virus displacements. Because virus motion with relatively long displacements (>0.3 μm/s) occur rarely, the tail of the function, which is the frequency distribution of the long displacements, fluctuates irregularly. ECCDF is suitable for analysis of such noisy tails.

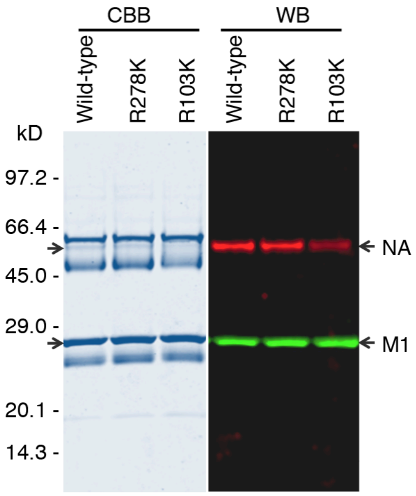

Supplementary Fig. S2. NA expression of wild-type and mutant PR8 viruses. Representative blots of viral proteins separated by SDS-PAGE (CBB) and NA proteins detected by western blotting (WB) are shown. The corresponding relative NA expression levels evaluated by the fluorescence intensities of NA bands and standardised by M1 band intensities are summarised in Table 1.

Supplementary Tables

Supplementary Table S1. Affinities of different receptors for their specific ligands prior to endocytosis.

Ligand (Virus)	Receptor	Dissociation coefficient (M)	Reference
Hemagglutinin (Influenza virus)	Sialyloligosaccharide	$1.4–6.5 \times 10^{-3}$	Sauter 1989[11] Sauter 1992[12]
G protein (Vesicular stomatitis virus)	LDL receptor family	10×10^{-9}	Finkelshtein 2013[13]
E2 protein (Semliki Forest virus)	MHC class I	40×10^{-9}	Helenius 1978[14]
Transferrin	Transferrin receptor	7×10^{-9}	Dautry-Varsat 1983[15]
Low density lipoprotein	LDL receptor	3.2×10^{-9}	Wathne 1989[16]
Epidermal growth factor	EGF receptor	$1–10 \times 10^{-9}$ (95–98%) $10–100 \times 10^{-12}$ (2–5%)	Lax 1989[17]
Insulin	Insulin receptor	$50–75 \times 10^{-12}$	Cuatrecasas 1971[18]

Supplementary Table S2. Lateral diffusion of receptors involved in endocytosis.

Receptor	Cell	Diffusion coefficient $\times 10^{-2}$ ($\mu m^2\ s^{-1}$)	Mobile fraction (%)	Reference
Transferrin receptor	Mouse epithelial cell	0.78<br>0.44	65<br>24	Kusumi 1993[22]
LDL receptor	Human fibroblast	0.05–0.2 (10 °C)	<20	Barak 1982[23]
LDL receptor (Internalization-defective mutant)	Human fibroblast (J.D. cell)	0.45 (28 °C)	60	Barak 1982[23]
EGF receptor	Mouse fibroblast	0.1–1.0	<10	Schlessinger 1978[24]
Insulin receptor	Mouse fibroblast	0.1–1.0	<10	Schlessinger 1978[24]
Influenza virus				
-Aich2	Fetuin-coated glass surface	1.1	~100	*This study*
-PR8		0.72	~100	

Supplementary Movie Legends

Supplementary Movie S1. Influenza virus particle movement on a fetuin-coated glass surface. Green line indicates the Aichi2 virus trajectory. This video plays at 20× speed (scale bar = 1 μm).

Supplementary Movie S2. Movement of virus particles on a fetuin-coated glass surface. Coloured lines in the last frame indicate Aichi2 virus particle trajectories. This video plays at 100× speed (scale bar = 1 μm).

Supplementary Movie S3. Aichi2 virus particles on a fetuin-coated glass surface in the presence of zanamivir. This video plays at 100× speed (scale bar = 1 μm).

Supplementary Movie S4. Wild-type PR8 virus movement on a fetuin-coated glass surface. This video plays at 50× speed (scale bar = 1 μm).

Supplementary Movie S5. NA R278K mutant PR8 virus movement on a fetuin-coated glass surface. This video plays at 50× speed (scale bar = 1 μm).

Supplementary Movie S6. NA R103K mutant PR8 virus movement on a fetuin-coated glass surface. This video plays at 50× speed (scale bar = 1 μm).

Supplementary Movie S7. Virus particles on erythrocyte surfaces in the absence of zanamivir. The first frame is a bright field image of erythrocytes fixed to a glass surface. Virus particle brightness fluctuates and the virus is slightly out of focus, implying that viruses move in three dimensions along the erythrocyte surface. This video plays at 100× speed (scale bar = 10 μm).

Supplementary Movie S8. Virus particles on erythrocyte surfaces in the presence of zanamivir. The first frame is a bright field image of erythrocytes fixed to a glass surface. This video plays at 100× speed (scale bar = 10 μm).
